# Hope is Not a Strategy: Using Robust Real-World Evidence to Make Better Clinical Development Decisions

**DOI:** 10.1007/s43441-025-00822-x

**Published:** 2025-07-18

**Authors:** Nicolle M. Gatto, Ulka B. Campbell

**Affiliations:** 1 Aetion, Inc., 5 Pennsylvania Plaza, New York, NY 10001 USA; 2 Department of Epidemiology, Mailman School of Public Health, Columbia University, New York, NY USA

**Keywords:** Real-world data, Real-world evidence, Clinical development

## Abstract

Clinical development delays and failures do not serve public health. Reliance solely on expert opinion or historical patterns rather than evidence from representative, real-world point-of-care data about the indication results in suboptimal trial design, missed opportunities, and uninterpretable findings. Investment in real-world evidence (RWE) generation to build a deep, comprehensive, and current understanding of the characteristics, care, and outcomes of the indicated population is essential to improving clinical development decision making. Despite the recognized value of RWE, this evidence generation is not done systematically. Here we make integrated RWE generation more compelling and practicable by addressing concerns we have heard from biopharma leaders and, for emerging RWE leaders, providing a blueprint for designing real-world studies in a phased approach that aligns with clinical development investment. Our work is intended to facilitate more widespread adoption of integrated RWE generation, beginning early in development, so that robust RWE is in hand at the right time for evidence-based decision making by the sponsor, regulators, and payers.

**Supplementary Information:**

The online version contains supplementary material available at 10.1007/s43441-025-00822-x.

## Introduction

The high cost, length, and failure rate of clinical drug development are bad for public health. Program delays due to regulatory strategy changes and trial completion challenges, and program failures due to reasons other than unfavorable benefit-risk profile, can block patient access to beneficial treatment. The high costs of development are eventually passed to patients and payers. To address these challenges, there has been substantial focus on ways to improve the efficiency of clinical development, such as adaptive clinical trial design, decentralized recruitment and consent, and more targeted site selection. Alongside these innovations, and based on our experience as epidemiologists working within and for the biopharma industry for 25 years, it is clear to us that the systematic integration of insights and evidence derived from real-world data (which we refer to as RWE) within development must be part of the solution. However, systematic integrated RWE generation is not standard practice—we want to change that.

## Perspective

Development teams should start early to develop a deep, comprehensive, and current understanding of the indication based on representative, real-world point-of-care data (rather than based primarily on expert opinion, historical patterns, or information on an overlapping or broader indication); and build this understanding using a systematic, phased approach with RWE investment aligned with clinical development investment. While this is intuitive to industry-experienced epidemiologists, in our experience it is often done piecemeal, too late, or not at all. We think this is largely due to budget constraints and enterprise structures that do not facilitate adequate and efficient access to the relevant expertise within the organization. Below we address these issues to make systematic integrated RWE generation more compelling and practicable, with two types of readers in mind. The first is the senior biopharma decision maker (e.g. Head of Research and Development, Chief Executive Officer or company board member) who may not fully appreciate the value of RWE, including the difficulties caused by not having it and the positive outcomes when it is in hand, and is in the position to commit the resources necessary for systematic RWE generation. The second type of reader is an emerging biopharma epidemiology or RWE leader who is trying to build or grow an organization and needs to articulate the return on investment, and how and when to generate RWE.

### To the Senior Decision Maker

Although there is a lot of enthusiasm around the use of real-world data (RWD) in lieu of a randomized controlled trial (RCT) for regulatory approval, FDA guidance is clear that this approach is acceptable in only limited circumstances—when an RCT is not feasible, and the validity and trustworthiness of real-world study results are clearly demonstrated. Successful uses have generally involved rare, deadly diseases with high unmet need, for which a RWD-based pivotal study was based on good understanding of the disease condition, and evidence from this pivotal study along with strong confirmatory evidence was sufficient for approval [1–4].

However, there are other impactful and more universally applicable uses of RWE in regulatory applications, such as justifying a non-randomized trial design, demonstrating the representativeness of a trial population, and establishing natural history or providing a benchmark to support causal interpretation of trial findings [5]. These RWE uses are epidemiology basics: analyzing RWD to develop robust understanding of the baseline characteristics, treatment utilization including standard of care, and natural history of the indicated population. This research is critical for decision making by sponsors and regulatory authorities. In fact, much of the information needed for evidence-based decision making can only be derived from RWD analysis. While not the focus here, we note this information is also critical to build a treatment’s value story for successful commercialization, further increasing the return on investment.

Several authors have proposed how RWE can support clinical development (for two particularly comprehensive reviews see Dagenais 2022 CPT and Zhu 2023 CPT). Dagenais et al. [6] describe how RWD can be used to make portfolio decisions based on market size from incidence/prevalence, inform product development and target identification with linked omics data, and optimize trial design. Zhu et al. [7] review RWE applications relevant to clinical pharmacology and provide an insightful diagram demonstrating uses of RWE across the development timeline. While some companies have woven RWE into their integrated evidence plans, much of industry still struggles to make systematic investments. Common blockers to early, systematic RWE generation are addressed below.

One concern is that the required investment is too big with uncertain return. If we consider the cost to bring a drug to market (~ $160 million dollars on average, but at times > $2 billion) [8], the RWD studies we’re proposing will generally cost < 3% of this budget. This relatively small RWE investment should be made for every program, starting early and phased with clinical development so that the RWE spend tracks with the overall program spend. RWE investments will pay off over and over, especially when a sponsor has multiple development programs for the same indication.

Early and robust understanding of the indication means having data to support informed, evidence-based decisions about program design and regulatory pathways, and to justify those decisions in regulatory filings. When this work is done proactively, RWE may also provide an insurance policy in case of trial failure. Lack of this understanding results in a disease and treatment narrative that is based on belief, and often causes a scramble to get data to substantiate those beliefs—at great expense and risk of being surprised when the data contradict beliefs.

We have seen many instances of what can go awry when the sponsor  has not done this critical “homework”. For example, one sponsor relied on the pivotal trial design used to support the approval of another treatment for a similar indication but did not monitor standard of care which had changed appreciably, making the trial more difficult to implement and risking less compelling results. Another sponsor relied on evidence of surrogate endpoint validity from a population that is related to, but not the same as, the proposed indicated population, which was not accepted by FDA and delayed accelerated approval. A third sponsor proposed a real-world study as a pivotal investigation for a label expansion based on its belief that a randomized trial was infeasible due to widespread off-label use, but was requested by FDA to provide an evidence-based justification. Subsequent analyses of real-world treatment patterns showed that off-label use was less extensive and confined to patients with unique disease characteristics seen in highly specialized centers, which undermined the arguments of RCT infeasibility and real-world study feasibility and led to delayed pivotal evidence generation.

A related challenge stems from the structure of the organization. Biopharma sponsors experience frequent organizational changes, which often involve oscillations between centralizing and decentralizing RWE functions (e.g., epidemiology, outcomes research) and remit changes. These changes cause significant disruptions in the relationships among these functions and with their internal stakeholders, such as development teams, leading to missed opportunities for collaboration and efficient and maximally impactful RWE generation. A fuller analysis of organizational changes and their implications for return on RWE investment is outside the scope of our paper. But ideally, organizations are set up to facilitate easy access to expertise in epidemiologic study design. This expertise ensures that results from RWD analyses are interpretable and reliable because key variables (such as clinical history, comorbidities, severity, treatments and outcomes) are validly measured, and the data include representative patients, an appropriately large sample size, and have clearly defined denominators. Furthermore, ideal organizational structures facilitate early collaboration among RWE functions and with internal stakeholders so that all needs across the product lifecycle can be anticipated. For example, an epidemiologist can work with the clinical and regulatory leads in reviewing insights from treatment utilization analyses refreshed throughout development to inform trial design; working with an outcomes researcher at the outset ensures understanding of commercially important markets so that these analyses are set up for efficient refreshes post-launch to monitor and understand uptake of the newly approved product.

Another common challenge is uncertainty about when to start generating RWE or how to use it. For decades, industry epidemiologists have been using RWE to support orphan drug applications, to contextualize potential safety signals in clinical trials, and to address post-approval requirements. Beyond these uses, many sponsors are generating RWE in a targeted way (i.e., piecemeal) rather than systematically. In our experience, RWE functions rarely have their own research budgets and thus expend considerable effort to secure funding for each proposed study; this forces choices among studies that are equally important for development decisions. This piecemeal approach is both insufficient and inefficient. To have the most positive impact, real-world studies of the characteristics, treatment patterns, and natural history (as described in the next section) should be done for every indication targeted by a sponsor’s clinical development pipeline. For example, we recently worked with a sponsor to obtain data on real-world treatment patterns and outcomes within the indicated population as part of an evidence-based argument to FDA that an RCT is likely infeasible and an alternative pivotal design warrants further exploration; FDA agreed and this exploration work is ongoing. Starting early in development is essential—it facilitates informed decisions about trial design and regulatory pathways, and illuminates the need for more in-depth and time-consuming real-world data collection. When primary data collection is needed, for instance, such as for some rare diseases or diseases that are managed using scale-measured functional outcomes, these investments will be most valuable when started in Phase I.

To maximize the return on investment in RWE generation, we suggest:

- Including an experienced epidemiologist on the team responsible for decisions on research and development investment, strategy and likelihood of success
- Assigning the same epidemiologist (or the same function) to support the program beginning in Phase I and continuing through end of exclusivity, with as few hand-offs as possible (similar to how many companies assign Safety Clinicians)
- Incorporating a standard set of descriptive epidemiological studies into every integrated evidence plan
- Setting aside 3% of the budget for RWE generation on the indication (does not include RWE as pivotal evidence or post-approval studies)

### To the Emerging RWE Leader

RWE supports clinical development decisions by addressing four aims: (1) characterizing the epidemiology of the disease or condition the medicine or vaccine is intended to treat or prevent (i.e. the indication), and the baseline demographic, clinical and sub-clinical characteristics of the indicated population; (2) within the indicated population, characterizing patterns of clinical care, including treatments, as well as biomarker and other assessments used to monitor disease progression and outcome risks; (3) within the indicated population, characterizing sub-clinical and clinical outcome risks and disease progression over time (i.e., establishing natural history or clinical course of the disease under currently available therapies and extent of unmet medical need), including variation across subgroups of severity or other patient characteristics; (4) in a trial-similar population (i.e., a subset of the indicated population that is as similar as possible to the pivotal trial population), characterizing safety and effectiveness endpoints.

For Aims (1) through (3), the analysis goal is to describe and understand. For Aim (4), the analysis goal is to support a causal interpretation about treatment effects. While some parts of this work can be done in parallel, there is a natural progression in accumulating evidence. We first need to understand how to define the disease, then understand the characteristics of the indicated population and identify initial subgroups, and then understand treatment utilization and assessments used for disease monitoring (Aims 1 and 2). With that understanding, we can define and estimate the risks of outcomes, including surrogate and clinical effectiveness endpoints as well as potential safety events, and characterize how patients progress through disease states. Outcome risks are estimated overall and within subgroups to understand their patterns, with the goal of identifying or confirming subgroups characterized by within-group homogeneous outcome risk and across-group heterogeneous outcome risk (Aim 3).

Findings from Aims (1) through (3) are necessary to design and implement comparable real-world contextualizing cohorts to support causal interpretation of pivotal trials (Aim 4). In special circumstances, this descriptive work is also essential to rationalize, design, and implement a causally interpretable real-world study as part of a pivotal investigation such as an externally controlled trial (Aim 5). Generally, we recommend starting Aims 1 and 2 in preclinical/early Phase I, Aim 3 in late Phase I/early Phase II, and Aim 4 in late Phase II.

**Figure 1:**
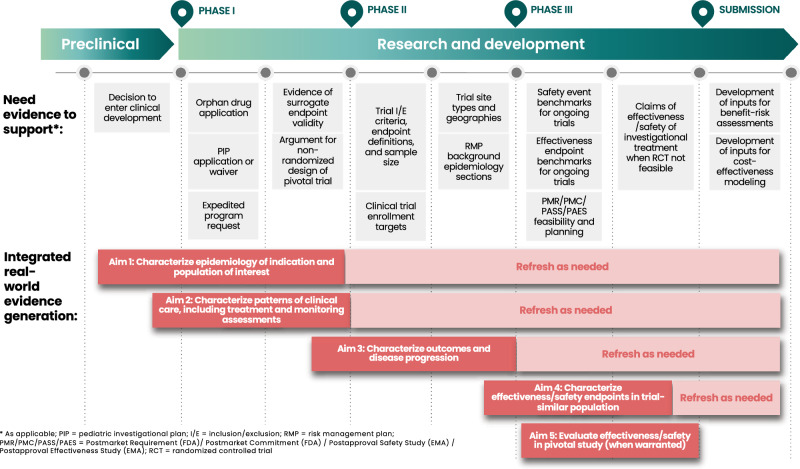
Integrating RWE into clinical development: needs and RWE evidence generation strategy.

Figure 1 shows how this RWE generation strategy serves the needs of clinical development. Figure 2 provides a blueprint for designing the real-world studies under this strategy, including the questions that must be answered for each development need and the specific real-world study objectives that align with these questions. See Table S1 for details on data points (A)–(L).

**Figure:**
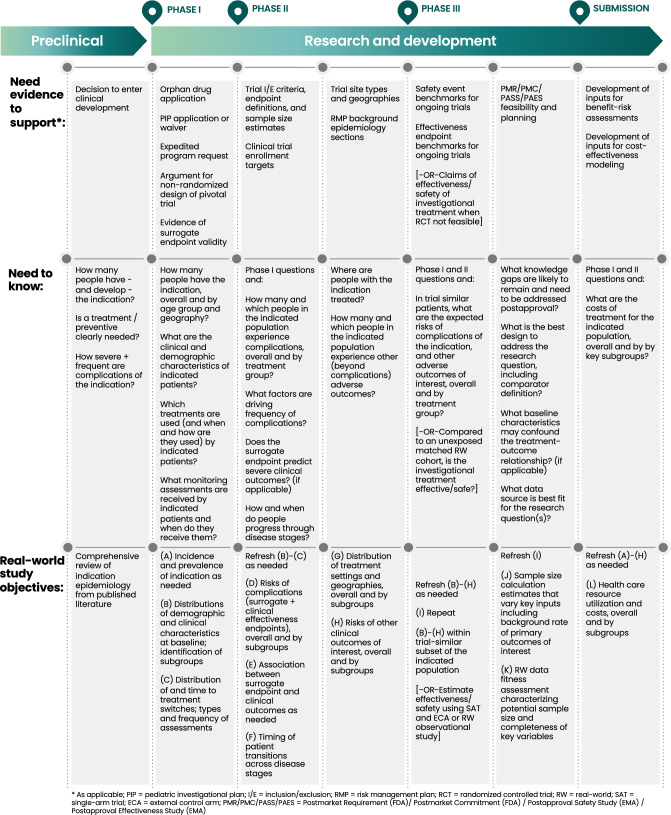
Integrating RWE into clinical development: Blueprint of real-world study objectives.

## Conclusion

A clinical development strategy based largely on projections, belief, and expert opinion about the indication is not likely to be successful. Despite advice to use RWE for evidence-based development decisions, industry has not widely adopted a systematic approach of integrated RWE generation. We have seen the ramifications of this resistance and have been asked for advice by many development teams struggling with a flailing strategy. In each of these cases, the sponsor received positive regulatory feedback after obtaining and applying relevant insights from real-world studies of the characteristics, treatment patterns, and outcomes of the indicated population, and shifting the strategy accordingly. Return on RWE investment can be maximized by systematically integrating RWE generation into early development and involving an experienced epidemiologist to ensure decision making and filings are supported by reliable information on the indication. Having robust RWE in hand at the right time not only supports evidence-based decision making by the sponsor, but regulators and payers as well, and helps ensure drug development serves public health.

## Supplementary Information

Below is the link to the electronic supplementary material.

Supplementary file1 (DOCX 14 kb)

## Data Availability

No datasets were generated or analysed during the current study.
