# Supplementary material for: Hope is Not a Strategy: Using Robust Real-World Evidence to Make Better Clinical Development Decisions
Source: Ther Innov Regul Sci. 2025 Jul 18;59(6):1288–93. doi: 10.1007/s43441-025-00822-x (PMC12579674; doi:10.1007/s43441-025-00822-x)
Supplement: Supplementary file 1 — Supplementary file1 (DOCX 14 kb) [file 43441_2025_822_MOESM1_ESM.docx]

S**upplementary Appendix**

**Table S1.** A blueprint guide for generating RWE on the indicated population to help optimize clinical development decision making

| **When** | **What** | **Why** |
| --- | --- | --- |
| **Prior to Phase I** | Comprehensive synthesis of peer-reviewed and gray literature to understand the basic epidemiology of the indication including incidence, prevalence, morbidity, mortality, comorbidities, complications, and treatments, including the number of people untreated and identifying the standard of care (if one exists) | To determine whether to enter clinical development for a particular treatment (or preventive, like a vaccine) and indication, the development team needs to understand how many people have and develop the indication (or would be eligible for the preventive), how severe and frequent the complications of the indication are under currently used treatments/preventives, and whether a new treatment / preventive is clearly needed. A comprehensive literature review can help answer some of these questions. |
| **Early Phase I** | Within the specific indicated population, in each region of interest, conduct studies to:  (A) provide more information about the incidence and prevalence of the indication. At times, it is necessary to conduct a study to confirm or address gaps in published estimates – the team epidemiologist can advise when this is needed.  (B) understand the distributions of baseline demographic and clinical characteristics, and important patient subgroups  (C) understand clinical care of indicated patients, including the distribution and timing of treatment switches, and types and frequency of monitoring assessments received (Note: Marketing data such as total sales may be helpful to understand regional treatment differences, but are not useful standalone.) | Conduct studies to confirm literature findings, fill in knowledge gaps and ensure timely evidence. Even with comprehensive review of the literature, there are often many important knowledge gaps. Peer-reviewed publications may be out-of-date and are rarely conducted within the specific indication of interest in each major region of interest (i.e., countries or regions in which the company expects to seek approval/conduct trials).  Understanding subgroups of the indicated population with similar demographic / clinical characteristics is important for identifying subgroups with similar baseline risk of important clinical outcomes in subsequent steps. Data on assessments used to monitor indicated patients are critical to operationalizing outcome definitions in subsequent real-world studies. In general, robust information on the specific indication on populations within regions of interest is essential - data on adjacent populations are generally not sufficiently useful. |
| **Early Phase II** | Refresh (B)-(C) if a long time period has passed since initial analyses or there are known changes in the diagnosis, epidemiology or clinical care of the indication.  Conduct studies to:  (D) estimate the risks of complications (surrogate and clinical effectiveness endpoints) overall, and within key subgroups such as disease severity and standard of care treatment groups  (E) demonstrate the relationship between a surrogate endpoint and measures of clinical benefit. For programs intended to submit for approval based on a surrogate endpoint as a measure of benefit, it is necessary to demonstrate the association between the surrogate endpoint and the major clinical outcomes of the condition - this can be done using epidemiology studies or prediction analyses.  (F) understand the timing of patient transitions across severity stages | Baseline characteristics and treatment utilization of the indicated population may change with evolving environmental conditions and clinical care practices. Understanding of the current state is essential to designing feasible trials and setting appropriate diversity targets for trial enrollment. For example, if a long time has passed since (B)-(C) have been obtained, trial entry criteria may need to account for new use of other treatments (off-label or newly marketed).  Understanding of natural history, including transitions between disease severity stages, is critical for many aspects for trial design, including selecting outcomes as effectiveness endpoints, providing baseline outcome rates for sample size estimation, and determining entry criteria that facilitate achievable sample size and complete follow-up. These data are also critical in demonstrating unmet need for expedited program requests. FDA guidance^1^ suggests natural history studies are needed for rare diseases and that in some cases, these studies may need to begin early (even before an investigational drug has been identified).  Although there may be some existing evidence of surrogate validity, getting more evidence or more robust evidence may be needed. However, for conditions that are not well studied, we recommend generating this evidence in Phase I.  For ultra-rare conditions with no available treatment, however, it may be beneficial to conduct this research during pre-clinical development.^2^ Because the patient population is so small, collecting natural history data generally requires primary data collection over many years to identify appropriate outcomes for measuring treatment benefit. However, it should be noted that in the context of ultra-rare conditions, it may still be difficult to derive robust RWE from RWD. In these cases, RWE may be able to provide a qualitative context for evidence from published (often, small single arm) studies or expert opinion. If there are concerns about the feasibility of a natural history study, we recommend discussing the concerns, considerations and options with FDA as early as possible. |
| **Later Phase II** | Overall and by key subgroups, conduct studies to:  (G) understand the distribution of treatment settings and geographies  (H) estimate risks of other clinical outcomes of interest, e.g. potential adverse effects associated with prior treatment | Optimized trial operations (e.g. successful site recruitment strategy) require understanding of the types of settings in which indicated patients seek care and the geographic regions with high concentrations of these patients.  Background adverse outcome risks/rates are needed for early risk management planning. |
| **Early Phase III** | For programs with a randomized pivotal clinical trial:  Refresh (B)-(H) if a long time period has passed since previous analyses or there are known changes in the diagnosis, epidemiology or clinical care of the indication.  (I) conduct studies (B)-(H) within the trial-similar subset of the indicated population. This generally involves applying the key clinical trial criteria - limiting to only the most critical pivotal trial inclusion criteria - to the real-world cohort to obtain a trial-like subset of real-world patients.  For programs in which randomization is infeasible, and other conditions are met,^4^ RWE may instead be used to estimate effectiveness or safety by providing an external control for a single-arm trial or for follow-on indications for which the treatment is used off-label, via a fully observational study. | Understanding of the current indicated population is critical to having clinically relevant contextualizing data for assessing representativeness of the patients and findings captured in the pivotal trial and trial-similar real-world cohort (see below).  Robust data on the baseline characteristics, treatment utilization, disease progression, effectiveness and other (safety) endpoints in the trial-similar subset of the real-world indicated population support pivotal trial interpretation in several ways. Natural history data, including rates of effectiveness outcomes, in the trial-similar subset can corroborate the outcome rates observed in the trial comparator arm, as suggested in FDA’s recent draft guidance on meeting evidence standards with a single investigation.^3^ Safety event rates in the trial-similar subset can provide contextualizing information for imbalances seen across trial arms, particularly for rare events where interpretation is very difficult. Baseline characteristics and treatment utilization data in the trial-similar subset are useful to understand why real-world outcome rates may differ from those seen in the trial. In addition to supporting the pivotal trial interpretation, the trial-similar subset of the real-world indicated population provides an “insurance policy” in case pivotal trial enrollment is lagging or trial completion looks unlikely. It may be possible to convert the pivotal trial to a single-arm trial and supplement the comparator arm with data from the trial-similar subset.  In these limited circumstances, the insights from RWD analyses conducted to date may be used to rationalize a non-randomized evidence generation strategy (e.g., small indicated population with high morbidity/mortality), and inform the design (e.g., sample size requirements, entry criteria, endpoint definition) of an external control arm and single-arm trial or a fully observational study as the pivotal investigation. In addition, the infrastructure of the RWD studies can provide the setting for conducting these studies. |
| **Later Phase III** | Refresh analyses among trial-similar patients (I) to align with key regulatory dates, and prepare for postapproval studies. The need for postapproval studies is typically driven by safety or effectiveness evidence gaps identified by regulators peri-approval and can often be anticipated by a well-integrated team epidemiologist during development of the risk management plan and application. Once the research question is specified, data from real-world cohorts are needed to support post-approval study preparation (J), such as identifying the appropriate comparator group and using the background rates of primary outcomes of interest in regions where the study may be conducted to estimate sample size requirements. Much of this information should be available from the studies conducted in Phases I and II but may need to be refreshed. Real-world data fitness assessment characterizing potential sample size and completeness of key variables (K) will also be necessary to rationalize real-world postapproval study data source choices. | Refreshed characterization of the trial-similar subset of the indication ensures comparable data are on hand to support interpretation of the results from the completed pivotal trial(s), including: assessing representativeness of pivotal trial population; corroborating outcome rates seen in the comparator arm; and contextualizing potential safety signals.  Having current data on hand facilitates a head-start on feasible post-approval study design, more efficient peri-approval agreement with regulators about commitments, and post-approval compliance with committed timelines. |
| **Periapproval** | Refresh (A)-(H) as needed to support commercialization needs, such as the design of comparative effectiveness studies and cost effectiveness studies. Additional real-world data on healthcare utilization and costs associated with the indication overall and by key subgroups will be needed. | Refreshed data on the indication facilitates efficient monitoring of uptake post-approval, including the proportion of indicated patients treated with the newly approved product and their characteristics, may be used to inform the design (e.g., sample size requirements, and definitions of population, comparator, and endpoint) of comparative effectiveness studies, and provide inputs for cost-effectiveness modeling studies. |

RWE = real-world evidence; FDA = Food and Drug Administration; RWD = real-world data

**References**

1. Draft Guidance for Industry: Rare Diseases: Natural History Studies for Drug Development. U.S. Food and Drug Administration (FDA), March 2019. (<https://www.fda.gov/media/122425/download>)
2. Hybrid Public Workshop: Natural History Studies and Registries in the Development of Rare Disease Treatments. Washington, DC: The Reagan-Udall Foundation for the FDA, May 13, 2024. (<https://reaganudall.org/news-and-events/events/natural-history-studies-and-registries-development-rare-disease-treatments>)
3. Draft Guidance for Industry: Demonstrating Substantial Evidence of Effectiveness With One Adequate and Well-Controlled Clinical Investigation and Confirmatory Evidence. U.S. Food and Drug Administration (FDA), September 2023. (<https://www.fda.gov/media/172166/download>**)**
4. Campbell UB, Honig N, Gatto NM. SURF: A *S*creening Tool (for Sponsors) to Evaluate Whether *U*sing Real-World Data to Support an Effectiveness Claim in an FDA Application Has *R*egulatory *F*easibility. *Clin Pharmacol Ther.* 114: 981-993 (<https://doi.org/10.1002/cpt.3021>)
